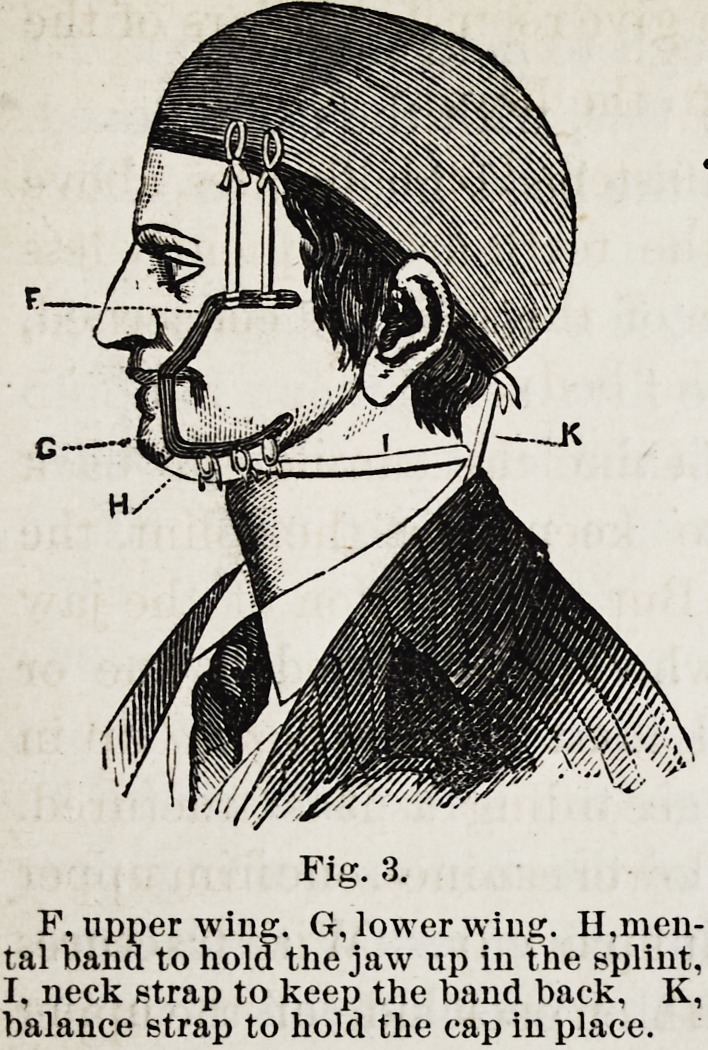# Treatment of Fracture of Lower Jaw by Interdental Splints

**Published:** 1868-06

**Authors:** Thomas Brian Gunning


AMERICAN JOURNAL

OF

DENTAL SCIENCE.

Vol. 11. THIRD SERIES—JUNE, 1868. No. 2.

### ARTICLE I.

### *Treatment of Fracture of the Lower Jaw by Interdental Splints.*

By Thomas Brian Gunning.

In the year 1840, when treating the first fractured lower jaw placed in my care, I found treatment by bandages, etc., unreliable. For, while the muscles tend to displace the bone, bandages frequently increase the difficulty; especially when swelling sets in through their pressure. They also, by interfering with the circulation, tend to prevent union. Teeth, loosened by the injury, are left unsupported, and the motions of the jaw, cheeks and lips painfully restricted.

Of the contrivances invented to supplement bandages, many were even more objectionable, and little improvement has been made in general treatment up to the present time. Having successfully used interdental splints, in many cases which had proved unmanageable under the usual treatment, I am convinced that they are superior to all other appliances.

When a well adapted splint is on the teeth and gum, the other parts around the bone are, to a great extent, a counter-support to the splint. Thus the broken jaw, together with any teeth loosened by the injury, is held securely in place, until the fractured bone is reunited and the teeth become firm. Meanwhile the motions of the jaw are in most cases unrestricted and the cheeks and lips always left free.

The best time to commence fitting a splint is immediately after the injury, if the condition of the patient will allow. If the fracture is old and has been treated by bandages, and there is much displacement of the fragments, with swelling of surrounding parts, it may be advisable to leave it *free* for several days.

When the fracture is not quite recent, pain and stiffness may prevent the patient from opening the mouth sufficintly to apply a splint, in which case the operator should force the jaw steadily downward with his fingers, assisted by wedges of wood, etc. This may be very painful to the patient at the time, but the movement of the parts, will be followed by great amelioration of the pain and stiffness. Hooks, forks and strings, applied to the teeth, will manage the fragments with less suffering to the patient than handling the inflamed muscles. The fragments of the jaw should be set and held by wire, pack-thread or silk, passed around the teeth. If the teeth are so formed that the ligature slips off, it may be carried through the gum with a needle. When a fragment of the jaw falls below the one next to it, a ligature of wire should be fastened around the neck of the lower tooth, two eyes being made by twisting the wire, before applying it. Another wire should be fastened around the neck of the elevated tooth, and both ends brought up on the side furthest from the fracture, over the crown, down through the eyes before mentioned, and then tightened until the bone is in place. Or the wire may be fastened to a tooth further back, and then passed over the crown, etc. On this principle, ligatures may be applied to the teeth laterally to bring the fragments into line. A jack-screw, furnished with points, forks and collars, is frequently necessary to extend the fragments, but in some cases it can be done by a piece of wood. The jack-screw should be made to turn by its centre, and the points, forks, etc., fitted into sockets, that they may be left still when the screw is turned. This instrument may be used across the mouth to keep out any back fragment that falls in, or more in front to extend oblique fractures. In fractures behind the canine when the back fragment comes forward over the front—being allowed to do so by the absence of teeth and the direction of the fracture—the jack-screw, with a point in the front fragment and a fork in the back one, will be found very useful in making extension. One fitted with hooks, to draw in the jaw by inserting both hooks near the external oblique lines, or in any required positions, will be found indispensable in some cases. A piece of hard wood forced in between those teeth which fall toward each other, and to which it must be fastened with a fine iron wire, will frequently give the needed extension. When the jaw is broken between the canines, with the fragments smooth and the parts around allowing them to go in any direction, there is frequently a front tooth absent, through the fracture, or by shedding, etc. In this case a piece of moderately hard wood may be fitted in the vacancy. It should be so wide that the adjoining teeth will press into its sides, when they are wired tightly. If this is well done the bone will be firmly set. Should the teeth in question need support, they may be wired to those adjacent.

An impression of the parts should be taken in pure yellow wax, warmed by *dry* heat. But in comminuted fractures there may be portions of the jaw and teeth for which plaster of Paris would be better, but it must be applied in sections. The wax should be applied in a mouth-cup adapted to the jaw. No. 4, splint is precisely what is required for this purpose. (Some useful hints may be found under that head.) If fracture should occur in a jaw without teeth, plaster would be much the best. It should then be applied in a cup to all parts of the jaw at one time. If possible (and it is rarely otherwise), an impression of all the teeth and gum, *properly set*, should be taken at one time. The wax in coming off will then draw or enlarge in the right places, and the plaster-cast from it will be precisely what is required to mould the splint, excepting the addition caused by the ligatures.

If the bone cannot be held in place, an impression of each fragment should be taken separately, and the casts from these impressions united by plaster in their proper relative positions. A cast of the upper teeth will sometimes guide in doing this. The united cast must be enlarged under those parts of the teeth which overhang. But when the pieces of the jaw can be held *nearly* in place, an impression of all may be taken at one time, the cast separated where necessary, and then adjusted as above.

By adopting this method, when there is little displacement, the jaw may be left unset until the splint is applied. When adjusting the cast, care must be taken that it is not made too small for the jaw and teeth as a whole, or for any tooth individually. There is little chance of getting it too large, as far as the teeth are concerned.

On February 12, 1861, I applied a "hard vulcanized rubber splint" to the fractured jaw of a seaman in the United States Naval Hospital, and from the vulcanite splints used by me since that time I have selected three which show all that is essential to hold any fractured lower jaw in place.

The fourth, a metal splint, is sufficient for the treatment of most cases, and can be applied by surgeons and country practitioners, who can also treat most cases of fracture with rubber splints, if assisted by the neighboring dentist. But a severe fracture may occasionally be met with, which will require either a specialist or an accomplished dental surgeon.

Fig. 1, represents the inner surface of a splint which incloses all the teeth and part of the gum of the lower jaw, and merely rests against the upper teeth when the jaws are closed. This splint is adapted to the treatment of all cases which have teeth on both sides of the fracture, except those with *obstinate* vertical displacement.

Fig. 1,

The holes marked A go through the top of the splint for the purpose of syringing the parts within with warm water during treatment. The dark round spots in all the cuts represent holes for similar purposes.

*The angles of the jaw tend outward, when the jaw is fractured through the body.* It is therefore necessary that the splint should go down and extend back as far on the outside as the muscles admit, especially on the short fragment, if there is much difference between them. The parts near the external oblique line are so formed that the splint can be fitted to them perfectly. The outer ends of the splint should be quite thick, so that they may be well rounded.

When the gum on the inside is so overhung by the back teeth as to afford but little bearing for the splint, the latter may be cut off, generally at or just below the edge of the gum, for there is rarely any tendency of the jaw to fall in at its lower border. The splint should not extend into the muscles unnecessarily in any part.

When the jaw is fractured in or near the front, the digastric and other muscles, inserted on the inside near the symphysis, draw the bone backward and downward. This splint neutralizes the first by holding the sides of the jaw *in*, which prevents the arch in front from falling back.

The tendency of the jaw to widen at the angles and to fall in at its upper border, so that the points of the canines approach each other, is also counteracted. The splint goes down about half way (on the outside) from the points of the teeth to the lower border of the jaw, and all the surfaces of the teeth and the outside of the gum are held by it, while the condyles and their interarticular cartilages are so far above the lower edge of the splint that their leverage prevents the sides of the jaw from being turned outward by the muscles inserted near the symphysis.

This must be effectual so long as the splint is down in its place; and even when the fracture is back of the canine, and the four pairs of muscles are acting upon the front of the jaw, there is little chance that they will draw it down out of the splint, as they act in concert with the elevator and other muscles attached to the bone, when the *splint is on and the jaw allowed to open and shut.*

There is also, in recent fractures, a roughness of surface, which prevents the fragments from moving when held close together. But if the fracture is so old that the fragments slide past each other, especially if the back one slants away and affords no support to the forward one, it *may* be necessary to hold the latter up by a screw passing through the splint into the canine or some other tooth, near the depressed end of the bone. That horizontal displacement which frequently follows fractures near the canine and lateral incisor teeth, in which the front of the jaw is drawn back by the muscles inserted near the symphysis, leaving the end of the short fragment in determined projection, and in which the treatment by bandage and ligature is not only useless but pernicious, is effectually overcome by this splint, without screws. A large proportion of all the fractures may be successfully treated in this way. When a *very* loose root or tooth is present, it may be advisable to remove it before application of the splint*. Rarely so before the impression is taken, as they are frequently of use in holding the jaw.

I have generally used this splint without any fastenings, but in children or even adults it is sometimes advisable to secure it by packthread, wire, screws passing into or between the teeth, or by the wings and band of Fig. 4.

Fig. 2. *In cases with obstinate vertical displacement*, the splint, in addition to fitting the teeth and gum of the lower jaw, must also inclose the upper teeth, as shown in the cut, where screws may be seen opposite both lower and upper teeth.

By this arrangement the fragments of the lower jaw are secured, not only relatively to each other, but also to the upper jaw.

---

* This is intended to refer to teeth loosened by disease, or loss of the alveolar, previous to the injury, not to those which may be loosened by it.

Fig. 2.

B, triangular opening, of which one side corresponds to the cutting edge of the lateral incisor, which tooth stood in the end of the fragment most displaced before the splint was applied. C, opening for food, speech, etc. D, channel for the saliva from parotid gland to enter the mouth, its fellow being seen on the other side of the splint. E', screw opposite lower canine tooth, head of the left screw being just discernible. E, head of screw opposite upper first molar tooth, end of its fellow being seen on the other side.

This splint is therefore adapted to the treatment of *all fractures back of the teeth*, whether in the body, the ramuses, or their terminations. In these cases the splint may be cut away in front, and extend across the roof of the mouth, when there are upper and lower back teeth to fasten to, and thus give as much room as possible to speak and eat through. Opening the teeth a quarter or three-eights of an inch would not have any bad effect on the position of the fragments, even if the jaw were broken through the necks of both condyles, as the parts near the fractures would move but little and the back of the jaw could be raised high enough to keep the broken surfaces in contact. Even if the neck of one side only were broken, the lower part could be kept firmly up against the fragment above. In fracture of the ramuses no difficulty would arise from this course. If a coronoid process were broken, this plan would give as good a chance for union as any. In fracture of the angle, this process would be likely to hold the parts in contact. If it did not, a wing could extend out from the splint and pass back from the corner of the mouth to hold a pad, etc., against the part requiring support; it could rest on the zygoma, or the mastoid process, if necessary.

In cases where enough of the front teeth are lost to afford room for food to enter, the jaws need not be opened more than will just give room for the rubber to pass through to hold the parts of the splint outside the teeth to the parts inside. A separation of a line would be sufficient, or *even less*, if any back teeth were absent to give room for pillars of the rubber to hold the upper splint to the lower.

As a rule, the splint should be fastened on both sides, above and below. Fractures back of the teeth are frequently less troublesome, so far as application of the splint is concerned, than those which are broken in the body.

When the body is fractured behind the canine, the back fragment requires no support to keep it in the splint, the muscles doing that effectually. But that portion of the jaw which includes the symphysis, whether separated on one or both sides from the parts behind, must be *firmly held* up in the splint by one or two screws, according as it is fractured. When the fracture is between the lower canines, one firm upper central incisor will hold the splint up firmly. With fractures in the *back* of the lower jaw, a tooth on each side of the upper jaw, back of the canines, would be sufficient for any case. Teeth which have lost much of their supporting alveolar will *bear great strain*, in the *direction of their sockets*, but the firmest teeth will suffer from slight lateral pressure; consequently ligatures are of little use, except temporarily. The thread must be removed from the screws on the ends which enter the teeth. The holes drilled to receive them should be from half a line to a line in depth, according to the size of the tooth. This will not injure the teeth, but they should be filled, however, after the jaw has united.

This splint can be made very thin, a shelly covering being all that is necessary in many parts. Openings should be cut in the sides where the absence of the teeth or separation of the jaws gives a chance for the saliva from the parotid glands to enter the mouth, otherwise it may overflow at the lips. Small openings should be made opposite particular teeth, to observe how the jaw stands in the splint. This is important in all splints.

Fig. 3.

F, upper wing. G, lower wing. H, mental band to hold the jaw up in the splint. I, neck strap to keep the band back. K, balance strap to hold the cap in place.

Fig. 3, shows the wings for cases *having no teeth in either jaw*—the ends of the wings within the month being imbedded in a vulcanite splint similar in principle to that of Fig. 2.

Wings made of steel may be quite light. They should have fine teeth along the edges where the band and tapes bear to prevent slipping, and small holes every half inch to hold the strings, lacing, etc. The arch of the wings should be high enough to give the lower lip room to go well up. The wings for each side of the jaw are in one piece, and the parts within the mouth pass back in the line of the upper gum. They are thinned down and pierced with holes, that the rubber in which they are imbedded may hold them firmly.

The tape strings pass from the cap inside and under the upper wings, then up between them and the tape lacings (see figure), which keep the strings from slipping, to the cap whence they started. The mental band passes up between the sides of the lower jaw and the wings where it is tied by the strings, which pass through the holes. (See figure.) The band is cut off to show this; but when worn it should be turned down on the outside and pinned just below the wings. The neck strap should be sewed to the mental band on one side and pinned on the the other, and worn tight enough to keep the band from slipping forward over the chin.

The jaw and splint are supported by the cap forward of its centre. This is counterbalanced by the elastic strap which passes from the back of the cap down around an unelastic and much heavier strap, extending across and fastened to the shoulders by elastic ends. The balance strap returns to the cap and is buckled tight enough to hold the jaw up. At night it may be slackened to do this, with the neck flexed. It slides on the shoulder strap as the head inclines to either side.

By this arrangement the splint is a resting place for the broken jaw, while the wings give firm attachment to appliances which hold the jaw up with the least possible pressure upon the external parts, as the wings need not press either against the jaw or the zygomas.

Should the band fail to keep a very depressed fragment in place, a metal loop may be fastened to the wings. From this, a metal point going through the soft parts could be brought to bear on any portion of the bone requiring firm support. (See Malgaigne.) But no external appliances, especially those which rest upon the muscles, can give the firm and comfortable support afforded by splints fastened to the teeth. Therefore, with suitable teeth in either jaw, the cap, or the mental band and corresponding wings, should be dispensed with.

When getting the articulation, or relative position of the jaws and teeth, it is necessary to bear in mind that the *position* of the lower jaw is *peculiarly* dependent upon the muscles attached to it. Neglect of this has caused great mistakes both in diagnosis and treatment, patients having been put to much suffering by the endeavors of surgeons to set fractures which did not exist, the displacements supposed to indicate them being the result of fracture in another part of the jaw—the latter being drawn out of shape by the muscles, etc. (suffering from laceration, contusion or severe swelling), and *thereby prevented from going into proper articulation with the upper jaw*, while the surgeon supposes that the ramus, or neck of the condyle, etc., is broken.

With only incomplete fracture, in which the bone retains its shape so perfectly that treatment is unnecessary, weeks or even months may elapse before the muscles are able to bring the jaw into place, so that the lower teeth will close against the upper, as before the injury. In fact, this inability may be present *without any* fracture of the bone.

These injuries are frequently aggravated by bandages, and the displacements increased and caused by them in the broken jaw, and also in its relation to the upper, are sometimes irremediable by any subsequent efforts, even in cases which correct treatment in the outset would have cured perfectly.

In consideration of these facts, it is important to discriminate between displacements which can be reduced by art and those which should be left to nature.

The fragments of the lower jaw having been set in their proper places relatively to each other, the whole must be put in normal relation to the upper jaw, *as near as the condition of the muscles and ligaments admit.*

If the jaw is allowed to move during treatment, it will generally go into place before the bone is firmly united.—When held still, it may not do so until some time after.

(Remarks upon displacement are given only so far as they are directly necessary to a proper application of the splints, and to an appreciation of their efficacy—the object of this paper. Correct diagnosis, however, is the foundation of proper treatment, and will be dwelt upon hereafter.)

Fig. 1, is the representative splint for the treatment of cases in the first class, or those in which the jaw is left free. Fig. 2, for the second class, or those in which the jaw is held still.

The articulation in each class is obtained by a method differing from the other. Consideration of these methods has been postponed until now, that they may be more easily understood. The reason for getting the articulation in different ways will be seen distinctly by recollecting that the fractures in the first class can be so *well* held together that the gutta-percha and wax have a firm resting place to carry them against the upper teeth. In the second class, however, it is frequently difficult, and occasionally impossible, to set the fragments in place, although it is desirable that *the splint*

should hold them precisely so as regards each other, and, as a whole, in the best possible position relatively to the upper jaw. Now, the upper jaw, being uninjured, affords a proper basis for the gutta-percha and wax. The lower jaw can, therefore, be pressed *carefully* up in place, and any fragment *specially* directed into the best attainable position in the wax. The wax, with its support of gutta-percha, may then be put upon the cast of the upper jaw, and the adjusted cast of the lower jaw placed in it precisely where required, as there is now a second opportunity to overcome any imperfections in the bite made by the teeth in the displaced fragments.

In the first class, a piece of dentist's gutta-percha should be warmed by *water*, and moulded to the plaster-cast of the lower teeth, etc. Upon this sufficient wax should be placed to give a bearing for the upper teeth and the proper thickness to the splint. When cold it must be placed on the *lower teeth*, and the jaws closed until the upper teeth press properly into the wax, then replaced upon the cast and trimmed into the shape required for the splint. The indentations made by the upper teeth should be cut down, so that only their points may touch the splint. The whole should then be set in a vulcanizing flask, to form the mould for the rubber splint.

But in the second class, as indicated before, the gutta-percha, etc., should be placed upon the *upper teeth* or *gum*, and the lower teeth or gum brought up in place. A case, however, is sometimes seen in which the articulation must be obtained in a radically different way.

When the upper teeth are so marked by the lower ones as to indicate the relative position of the fragments clearly, they do this for the jaws also; and by placing the adjusted cast against the upper cast, and setting them in an articulator, the normal relative position of the jaws, whether open or shut, may be obtained more accurately than from the mouth *in some fractures*. A model of the splint can therefore be made of gutta-percha. When quite cold from immersion in ice water, it should be put upon the upper jaw, and the fragments of the lower pressed up into it, to test the accuracy of the adjustments. This model might be used to form the mould for the splint without the original casts, if either were found incorrect, for the gutta-percha could be made to fit by a little heat and pressure. As a rule, it should only be used to set the casts.

This plan is less painful to the patient in extreme cases, as it avoids the setting of the fragments and taking the bite. But it requires considerable care, as allowance must be made for any altered condition of the fractured surfaces, and also for any inability of the fractured jaw to go into proper articlation with the upper.

The gutta-percha or wax, when taken from the mouth, should be placed between the casts representing the lower or broken jaw and that of the upper jaw, then cut into shape, the female screws, or the wings, imbedded, and the whole set in a suitable flask.

The nuts for the screws should be about an eighth of an inch square, and a little less than a line thick, thus giving sufficient length to the female screws in the centre. The nuts should be beveled down, inside and out, on three sides, but the fourth only down to the middle one of three gold strips, of which the nuts are formed. This strip, being left long, should be turned over a short distance from the nut and its edges notched—it will then act as a standard to hold the nut in place in the mould. Each nut must also have a piece of tough wood screwed into it. To set them in position, bore a hole in the plaster tooth exactly where the screws are to enter the natural teeth. Place one end of the wood into the hole with the *nut* against the plaster tooth, and bring the wax up close around it. In this way the other end of the wood will stand out and be imbedded with the gold strip in the plaster forming the mould, and the nuts held firmly while the rubber is packed.

(*To be Continued.*)

## Figures and Tables

**Fig, 1, f1:**
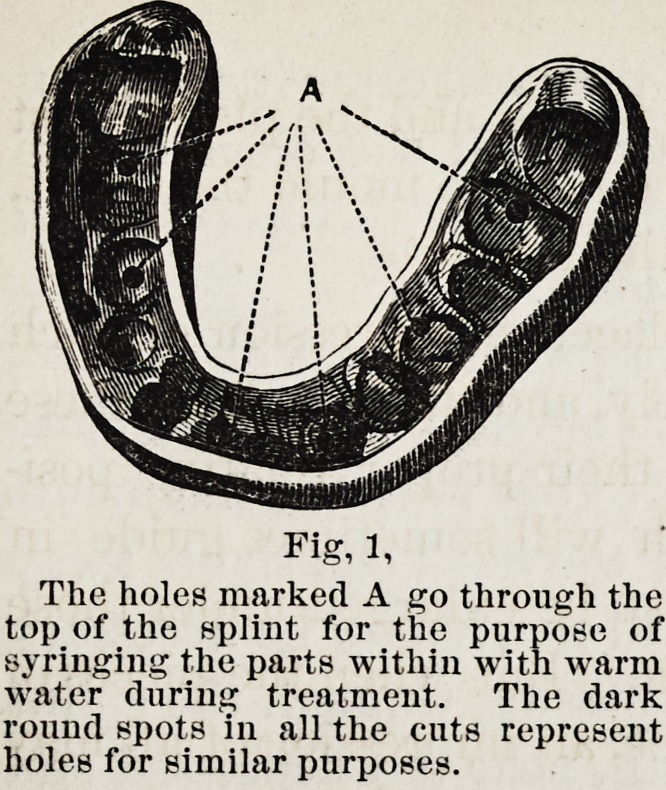


**Fig. 2. f2:**
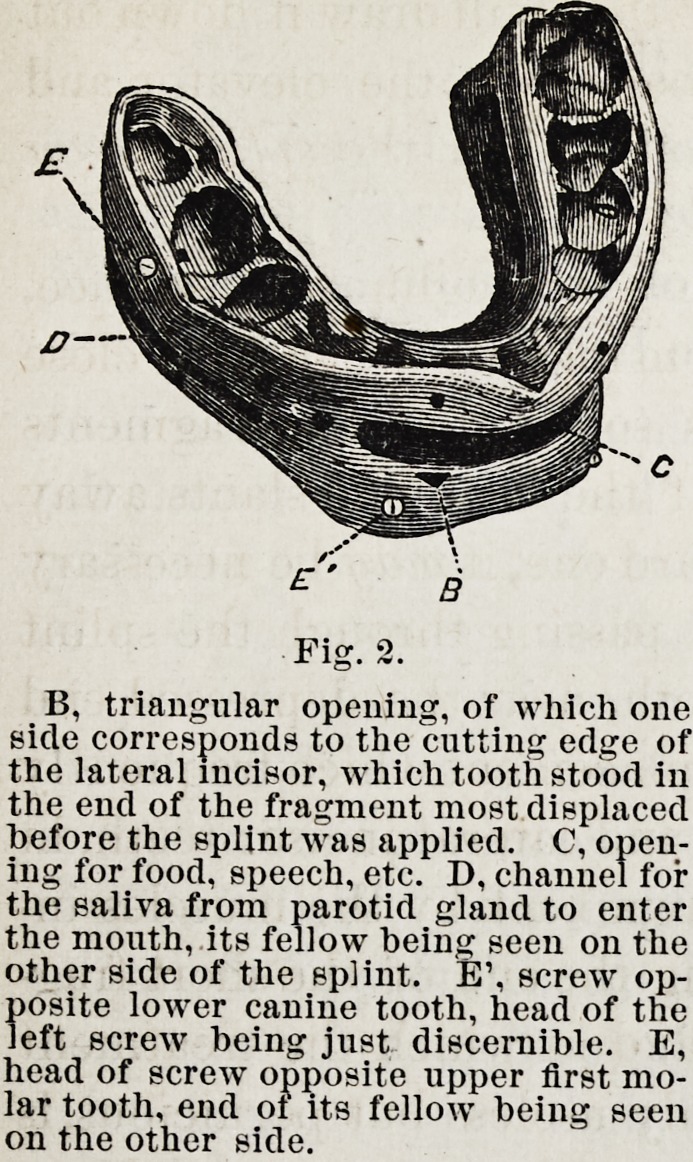


**Fig. 3. f3:**